# P69 - Development and evaluation of CARATKids, Control of Allergic Rhinitis and Asthma Test for children

**DOI:** 10.1186/2045-7022-4-S1-P124

**Published:** 2014-02-28

**Authors:** Daniela Linhares, Almeida João Fonseca, Miguel Luís Borrego, Mário Morais-Almeida

**Affiliations:** 1 CINTESIS, Faculty of Medicine, University of Porto, Porto, Portugal

## Background

CARAT is the first tool available to concurrently assess the control of rhinitis and asthma in adults. No similar questionnaire was, so far, available for children.

## Aim

To develop and evaluate the measurement properties of CARATKids, a questionnaire designed to assess the control of allergic rhinitis and asthma (ARA) in children with 6-12 years old with a previous medical diagnosis of ARA.

## Methods

### Development

1) A literature review for pediatric questionnaires on asthma and/or rhinitis control was performed;

2) A multidisciplinary working group held consensus meetings to develop CARATKids preliminary version;

3) A cognitive test was done in a cross-sectional, qualitative study, with face-to-face interviews of 29 children and their caregivers.

### Evaluation

Prospective multicenter validation study. In a 2 visits program scheduled 3-5 weeks apart, questionnaires (including cACT) and VAS were filled by children with ARA and their caregivers. Physicians blinded for questionnaire results assessed children’s ARA status. Logistic regression and reliability analysis were used to select the questions to be included in CARATKids final version; and discriminative properties, reliability and validity were evaluated.

## Results

### Development

A 17-item preliminary version with dichotomous (Yes/No) answer format accompanied by illustrative drawings was produced.

### Evaluation

113 children (44 female) and their caregivers were included in 11 centers. Children’s mean(sd) age was 8.8(1.9) years old, 48 had their asthma controlled and 37 had moderate/severe rhinitis. After item reduction, the final questionnaire has 13 questions (8 to be answered by the child and 5 by the caregivers). The internal reliability was 0.8; the correlations coefficients with external measures of control were higher than a priori predictions ranging between 0.516 (with physician assessment of rhinitis control) and 0.697 (with cACT).

## Conclusion

CARATKids, the first questionnaire to assess allergic rhinitis and asthma control in children, was developed and has adequate internal consistency, concurrent and clinical validity.

